# Baseline Levels and Temporal Stability of 27 Multiplexed Serum Cytokine Concentrations in Healthy Subjects

**DOI:** 10.1371/journal.pone.0076091

**Published:** 2013-12-12

**Authors:** Angelique Biancotto, Abigail Wank, Shira Perl, Wendell Cook, Matthew J. Olnes, Pradeep K. Dagur, J. Christopher Fuchs, Marc Langweiler, Ena Wang, J. Philip McCoy

**Affiliations:** Center for Human Immunology, Autoimmunity, and Inflammation, National Institutes of Health, Bethesda, Maryland, United States of America; Imperial College London, United Kingdom

## Abstract

**Background:**

Cytokines are humoral molecules that elicit regulatory function in immunologic pathways. The level and type of cytokine production has become critical in distinguishing physiologic from pathologic immune conditions. Cytokine profiling has become an important biomarker discovery tool in monitoring of the immune system. However, the variations in cytokine levels in individual subjects over time in healthy individuals have not been extensively studied. In this study, we use multiplex bead arrays to evaluate 27 analytes in paired serum samples taken seven days apart from 144 healthy individuals in order to assess variations over a short time period.

**Methods:**

Fluorescent bead-based immunoassay (Luminex) was used to measure 27 analytes in serum samples. Measurements were performed on matched samples from 144 healthy donors. To assess inter-plate variability, one arbitrarily selected serum sample was analyzed on each of the first ten plates as bridge sample.

**Results:**

Using the bridge sample, we showed minimal inter-plate variations in the measurement of most analytes. In measurement of cytokines from the 144 patients at two time points, we found that three cytokines (IL-2, IL-15 and GM-CSF) were undetectable and five analytes (RANTES, MCP-1, VEGF, MIP-1β and PDGF-BB) showed significant difference in concentrations at Day 0 compared to Day 7.

**Conclusions:**

The current study demonstrated higher variations in cytokine levels among individuals than were observed for samples obtained one week apart from identical donors. These data suggest that a serum sample from each subject for use as a baseline measurement is a better control for clinical trials rather than sera from a paired cohort.

## Introduction

Cytokines and chemokines are soluble molecules important for regulation of cell function in the innate and adaptive immune responses. Cytokines play a significant role in intercellular signaling in inflammatory reactions and hematopoiesis [1] whereas chemokines play a major role in chemotaxis in events such as inflammation or angiogenesis. Measurement of these immune mediators has become increasingly important as a means of understanding immune responses during the course of disease or infections, or in response to therapy and interventions. As these are soluble molecules, the detection and measurement of cytokines and chemokines generally relies on antibody-based capture techniques such as ELISA. Since the total number of known cytokines and chemokines is well over one hundred, single-plex assays for these are of limited utility when attempting to measure these as part of a broad profiling of immune responses in clinical protocols or trials.

In the past few years, increased interest in biomarker research has pushed forward the development of multiplexing tools. Biomarker studies allow researchers to examine and characterize a disease state in a non-invasive manner, and to correlate changes in the biomarker to disease status or therapy. While some biomarkers are critical for the early diagnosis of many chronic diseases, including heart diseases, diabetes, and cancer [1,2], others provide prognostic information or a better understanding of the pathogenesis of diseases. Many clinical studies involve the correlation between the presence and biological activity of cytokines and chemokines, as they are crucial for the understanding of many immunological functions [3].

Bead based multiplex immunoassay technologies to measure biomarkers have the capability of measuring up to 100 cytokines/chemokines simultaneously in a minimal amount of biological sample [4] and are becoming increasingly available. When conditions are carefully controlled, these multiplex assays can achieve similar results to those obtained by ELISA techniques [5]. Determining the accuracy and reliability of the detection of each analyte is key for the development of multiplex clinical tests and in using these in clinical trials. Our lab has examined potential sources of variation in these assays. For example, we found that quantification of cytokines varies greatly depending on which commercial kit is used for these determinations [6]. In recent studies, we compared the measurement of analytes in serum to those in plasma and found that these differ significantly for many analytes [7]. We have also looked at the impact of various anticoagulants on cytokine measurements in plasma [8] and again show that these can significantly impact the measurements of various analytes. Together these studies illustrate the need for strict uniformity in sample collection and preparation, as well as assay performance in order to obtain reproducible results. By understanding the limitations on sample collection and assay performance, questions could then be asked regarding the biologic variations that might occur in healthy subjects. This, in turn, can facilitate the design of better protocols for clinical trials in which cytokines are measured.

Little is known regarding the variations in levels of these biomarkers over time in healthy individuals. Interpretation of cytokine data in such trials is frequently made by comparing the mean levels of all individuals prior to intervention with the mean levels observed after intervention, such as vaccine or treatments [9–11]. Alternatively, measurements after intervention can be compared to measurements on samples from the same subjects pre-intervention. There is a paucity of data to indicate which approach is best. In the present study, we analyzed twenty-seven analytes in the serum of healthy individuals, collected at two time points seven days apart. Our primary objectives were to evaluate the stability in baseline measurements of cytokines in individual patients over a short period of time and to examine inter-individual variability of cytokine levels in order to better understand how inherent fluctuations might impact interpreting data from clinical trials. 

## Materials and Methods

### 2.a Samples

Volunteers were pre-screened with a questionnaire and physical exam and were excluded if they had signs of illness or autoimmune manifestations or were taking immune-modifying drugs. Serum samples were obtained at two time points seven days apart from each of 144 healthy donors. All samples were obtained between 7:30am and 9:30am workday mornings from fasting subjects. The donor ages ranged from 21-62 years, and the median age 27 years. The ethnicity of these subjects was: Caucasian (70%), Asian (14.7%), African American (2.1%), Hispanic (5.6%), and Middle Eastern descent (7.7%). Gender distribution was 65% women and 35% men. Written informed consent for all procedures and research collections was obtained on an Institutional Review Board-approved protocol (NCT01191853) in accordance with the declaration of Helsinki and reviewed by National Heart Lung and Blood institutional review board. Sera were extracted from 8ml SST tubes (Becton Dickinson, San Jose, CA) within 1-hour after blood was drawn and stored in liquid nitrogen until further use. 

### 2.b Laboratory methods

Using a 27-plex kit from Bio-Rad, all specimens were analyzed for IL-1β, IL-2, IL-4, IL-5, IL-6, IL-7, IL-8 (CXCL-8), IL-9, IL-10, IL-12p70, IL-13, IL-15, IL-17, eotaxin (CCL-11), IL-1Rα, basic fibroblast growth factor (FGF-β), granulocyte colony-stimulating factor (G-CSF), granulocyte-macrophage colony-stimulating factor (GM-CSF), IFN-γ, IFN inducible protein-10 (IP-10; CXCL-10), MCP-1 (CCL-2), macrophage inflammatory protein 1α (MIP-1α; CCL-3), MIP-1β (CCL-4), platelet-derived growth factor BB (PDGF-BB), RANTES (CCL-5), TNF-α, and vascular endothelial growth factor (VEGF). All Luminex assays were performed according to the instructions provided by the manufacturer (Bio-Rad). Serum samples were diluted four-fold, the minimal concentration allowed according to the manufacturer, and run in duplicate. Samples from the two time points from each patient were run in a total of 22 plates to measure serum cytokine concentrations in 144 healthy donors. Single lots of reagents were used for the entire study (plates and standards).

A minimum of 50 beads per analyte was acquired. Median fluorescence intensities were collected on a Luminex-100 instrument (Luminex, Bio-Rad), using Bio-Plex Manager software version 6.2 (Bio-Rad). Standard curves for each cytokine were generated using the premixed lyophilized standards provided in the kits. Serial 4-fold dilutions of the standards were run; S1 denoted the standard with highest concentration and S8 the standard with lowest concentration with S2, through S7 representing intermediate concentrations.

Cytokine concentrations in samples were determined from the standard curve using a 5-point-regression to transform mean fluorescence intensities into concentrations. Each sample was run in duplicate and the average of the duplicates was used as the measured concentration. In addition, a bridge sample, serum from a single healthy donor, was included in the first 10 plates to assess reproducibility and inter-plate variations.

 The lower and higher limits of detection were calculated for each cytokine by averaging the values for all 22 plates. To calculate the coefficient of variation (%CV) we divided the standard deviation of the mean by the mean and multiplied by 100. Analyses of the acquired data were then performed using Data Pro Manager 1.02 (Bio-Rad) and Prism Software version 6.0b.

### 2.c Statistical analyses

Statistical calculations were performed using Prism software version 6.0b. Data obtained with serum from one donor were considered as one experiment (n). When normality of distribution was proved using D'Agostino-Pearson omnibus test, a paired T test was used to compare the means; otherwise a Wilcoxon matched pair test was used. The analytes concentrations were compared by nonparametric analyses in which the mean or median was used to calculate significant differences. The significance level for the p values was set as p<0.05 and the actual p values were indicated for each series of experiments. In addition, the data were compared using the Spearman correlation test and when significant, the correlation coefficient, **ρ**, was reported. Median cytokine concentrations, interquartile ranges (IQR) and %CVs were also calculated.

## Results

### 3.1 Performance of the cytokine standards

All Luminex assays were performed using the same lot of cytokine standards in order to minimize inter-plate variation. The coefficients of variation of each dilution of the standards from all 22 plates and the means of those %CVs are presented in Table S1. The expected concentration and mean of the observed concentration of each cytokine are also in Table S1. Only two of the %CV values were higher than our desired acceptable value of 20%. These were the most concentrated standard (S1) for MCP-1 and MIP-1α. 

The limit of detection of the standards (lower and higher) generated by the standard curve was averaged, showing the dynamic range of each analyte; results are presented in Table S2 as mean ± standard error of the mean (SEM) and %CV (n=22). The lower and uppers limits of detection ranged from 0.8 to 71,526.7pg/mL, respectively. The greatest variation in the lower limit of detection was observed for G-CSF (25.4%), IL-4 (24.1%), RANTES (21.0%) and IFN-γ (24.7%). Two values for the %CV were above 20% for the higher limit of detection; MIP-1α (21.5%) and IL-6 (20.7%). All other %CVs for detection limits were below 20%.

### 3.2 Performance of the assay using a bridge sample

**Figure 1:**
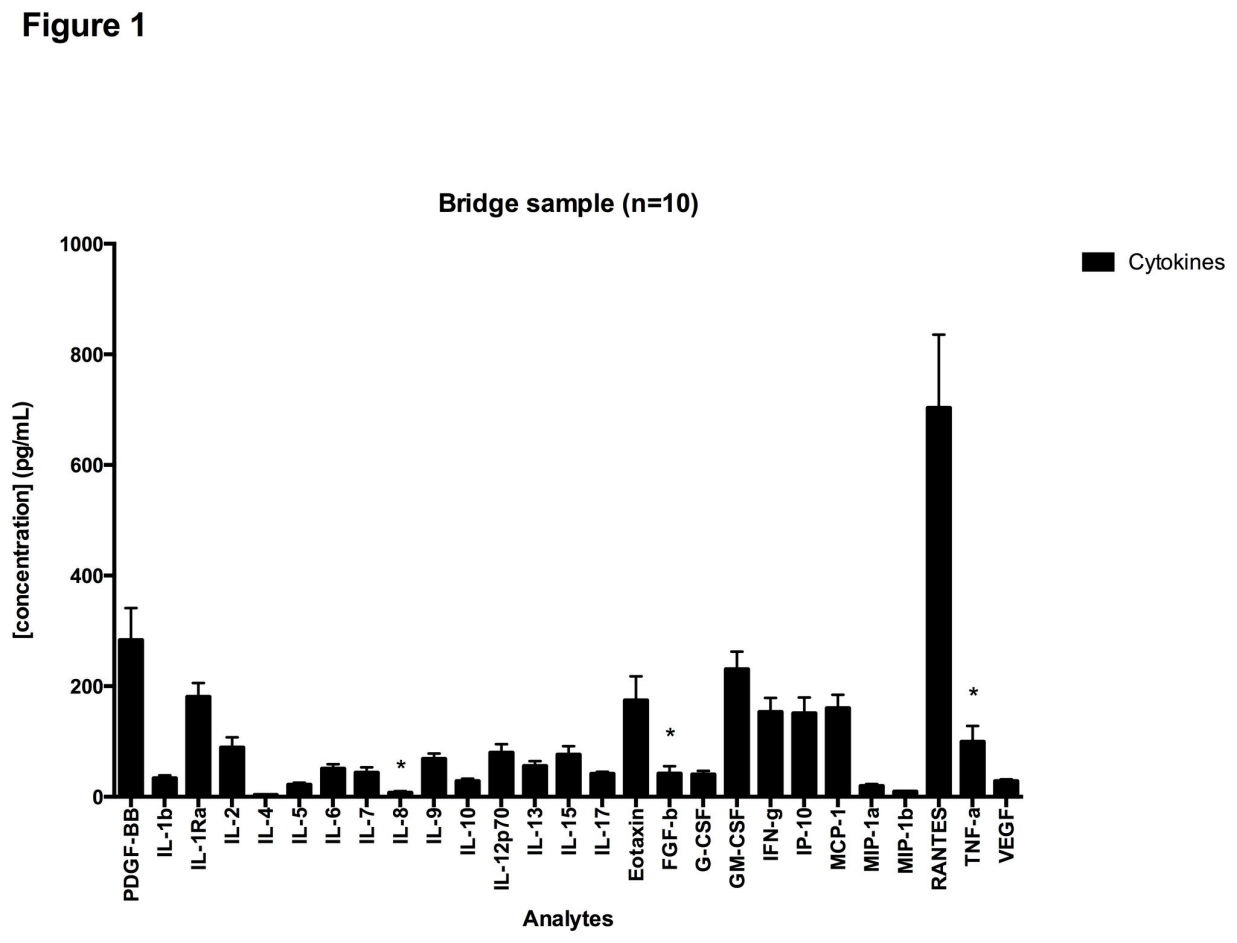
Cytokine concentrations in bridge sample. Results show mean concentrations of analytes for the bridge sample assayed in 10 plates with the bars representing the standard errors of the mean.

To measure inter-plate variation and to enable normalization of data across plates, we included one ‘bridge’ sample in the different plates. Each of the first 10 plates assayed included an internal control serum from a single healthy donor for use as a bridge sample. Figure 1 depicts the mean concentrations (n=10) with standard errors for each of the 27 analytes in the internal bridge sample (Table S3). The numeric values of mean concentrations, standard errors of the mean, and the %CV are summarized in Table S3. Eight out of 27 cytokines tested were with %CVs above 20%. The greatest variations were found in in IL-8 (36.0%), FGF-b (31.1%), and TNF-α (28.7%). 

### 3.3 Temporal variation of serum cytokines in 144 healthy donors

**Figure 2:**
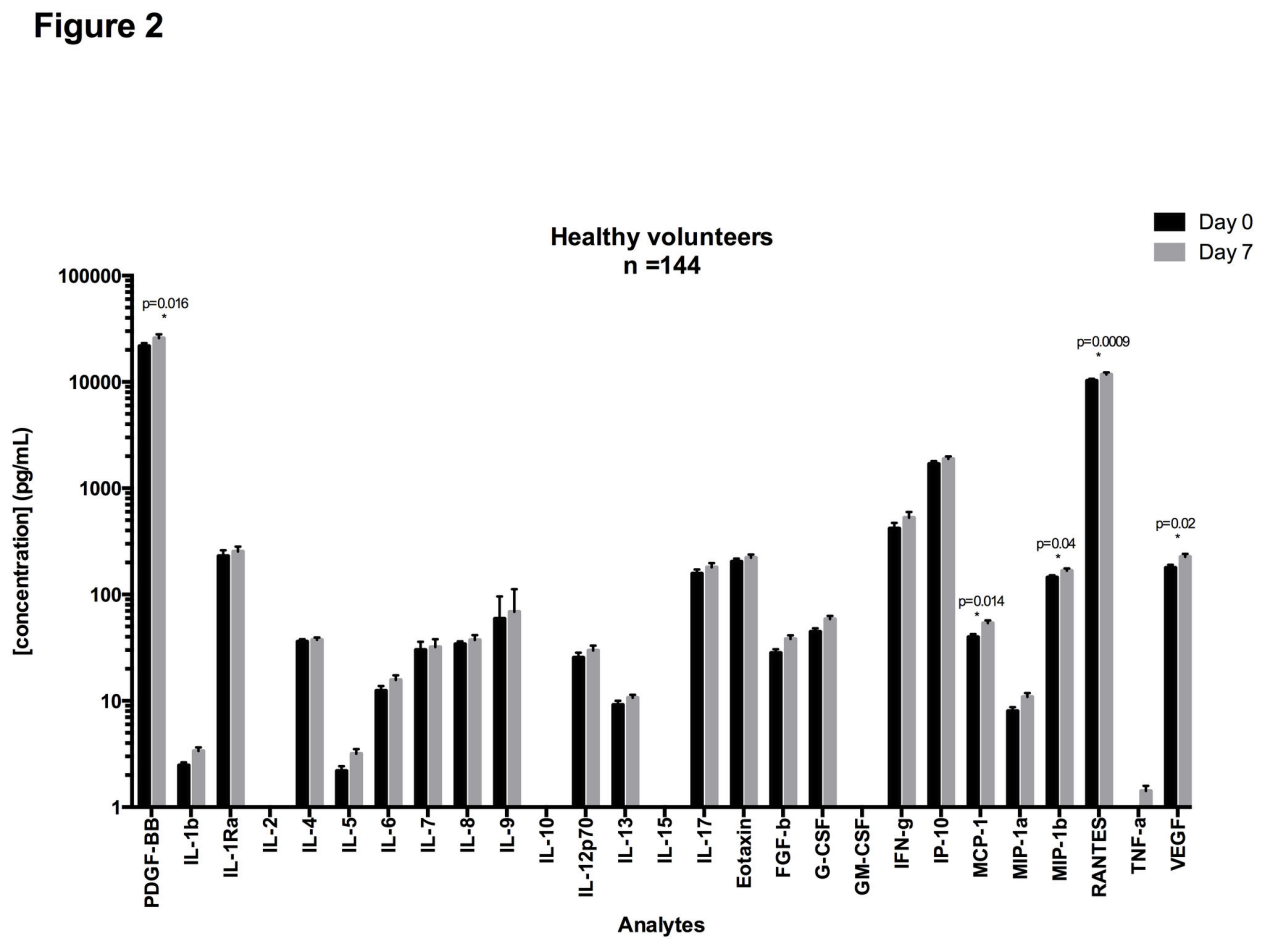
Mean concentrations for cytokines in serum samples at Day 0 and Day 7. Serum cytokine concentrations were measured on matched samples at Day 0 and Day 7. Paired T-tests were performed to measure the significance of the difference of the mean between Day 0 and Day 7. Cytokines for which %CV is higher than 20% are shown with “*”.

Serum from healthy volunteers was collected at two time points 7 days apart. Cytokine Luminex assays were performed using the 27-plex kit from Bio-Rad with the paired samples being run on the same plate for each subject. Figure 2 shows the mean concentrations and standard errors of the mean (SEM) from Day 0 and Day 7; the median concentrations are presented in Table S4 with interquartile ranges (IQR), and %CVs.

The median concentration of IL-2, IL-15, and GM-CSF were undetectable at either time point. The highest median concentrations detected were for PDGF-BB (25,863.34 pg/mL) and RANTES (11,740.25 pg/mL). 

Only 5 out of 27 cytokines showed significant differences in concentrations between Day 0 and Day 7: RANTES (*p=0.0009*), MCP-1 (*p=0.014*), VEGF (*p=0.02*), MIP-1β (*p=0.02*) and PDGF-bb (*p=0.03*). The remaining 19 analytes did not show significant differences in concentrations between Day 0 and Day 7.

**Figure 3:**
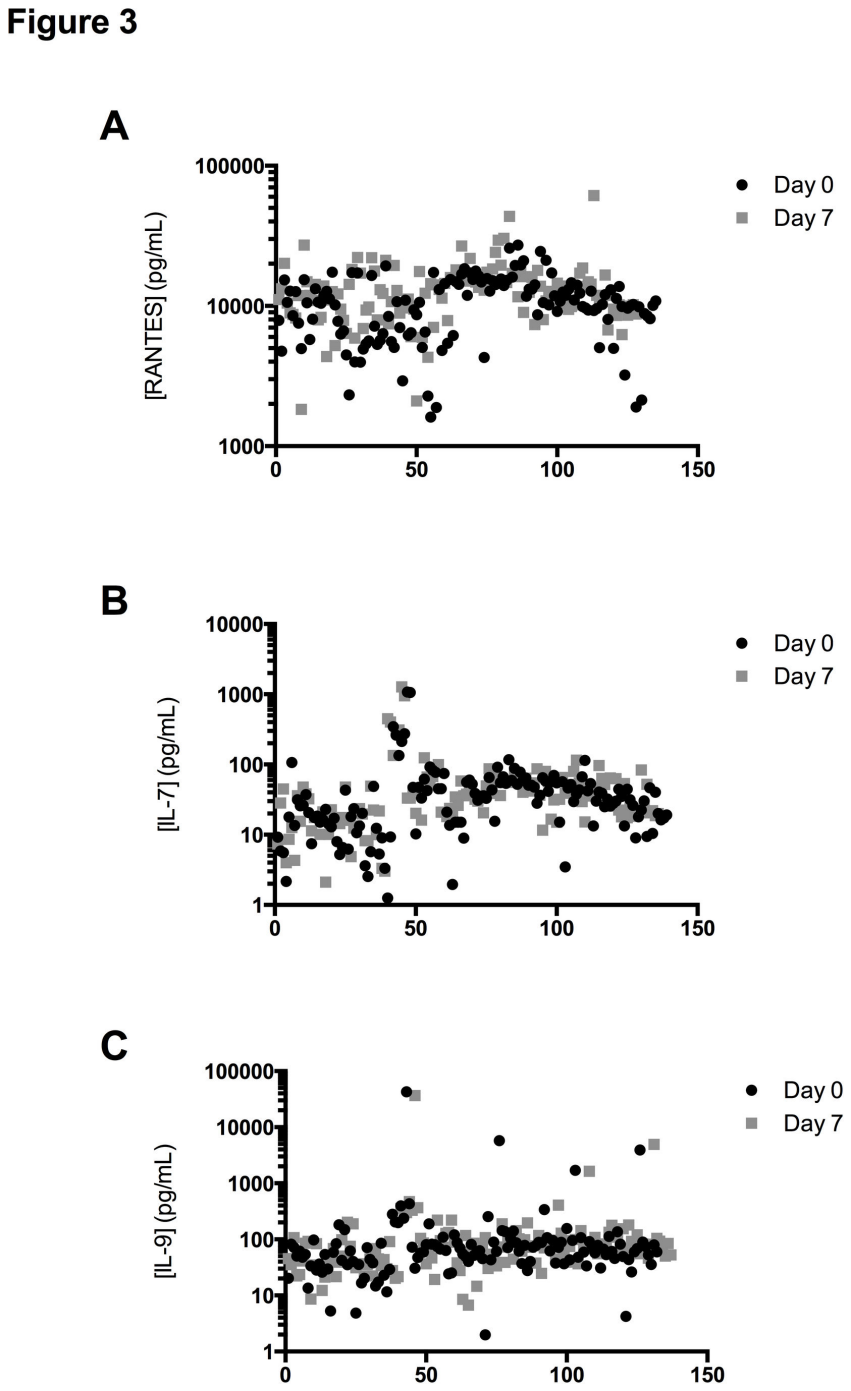
Concentrations for three cytokines in serum specimens measured at Day 0 and Day 7 for all 144 healthy volunteers. Serum cytokine concentrations were measured on samples at day 0 and day 7. Each data point corresponds to a matched measurement at Day 0 (black circle) and Day 7 (black square). These plots demonstrate 3 of the 27 analytes and display RANTES (A), IL-7 (B), IL-9 (C) concentrations.

Inter-subject variability at Day 0 and Day 7 was calculated using the %CV of all 144 donors (Table S4). On Day 0 the range of CVs was between 51.8% (RANTES) and 737.44% (IL-9) and on Day 7 the range was from 59.5% (RANTES) to 758.13% (IL-9), showing that a given cytokine had a similar inter-subject variability at Day 0 and at Day 7, even if their mean concentration was significantly different (RANTES). Figure 3 shows the concentrations measured for each donor at both time points, for cytokines with a high inter-subject %CV such as RANTES (Figure 3A), and for cytokines with intermediate %CV, such as IL-7 (Figure 3B) and high inter-subject variability such as IL-9 (Figure 3C).

For all cytokines combined, the inter-subject variability averaged 125% at Day 0 and 129% at Day 7. These data, together with the observation that only a few cytokines were significantly different in the paired matched samples, illustrates the importance of using an individual as its own control, as most of the cytokines are stable over time.

### 3.4 Serum cytokine concentration and age

To explore which factors may contribute to the high inter-subject variability evidenced by the %CVs, and the large interquartile ranges, we examined the correlation of age with cytokine concentration. We first calculated the Spearman coefficient of correlation ρ, and when significant for both Day 0 and Day 7, we calculated the linear regression R^2^.

**Figure 4:**
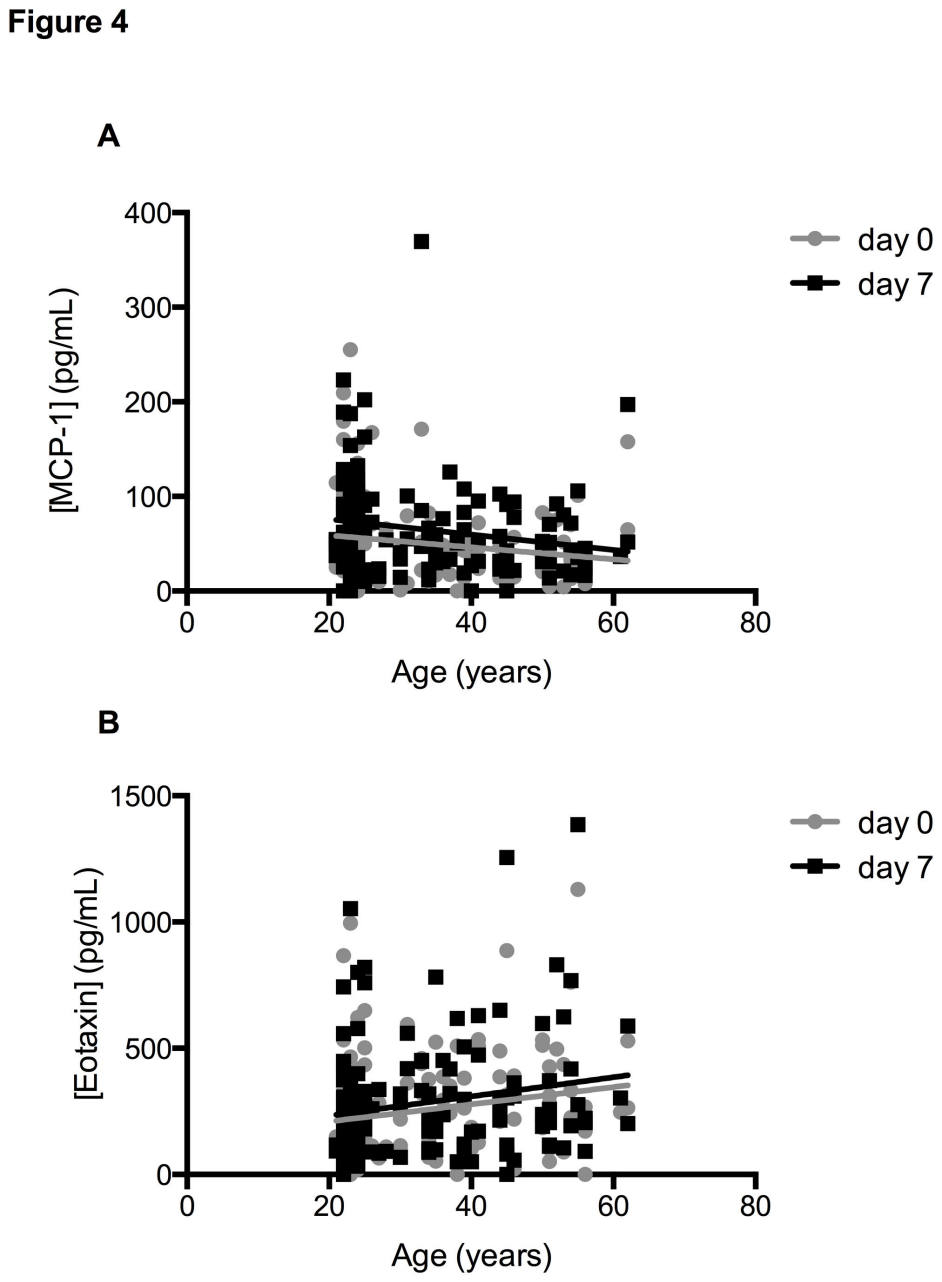
Correlation between cytokine concentration and age. Correlations of serum cytokine concentrations versus age were measured on samples at Day 0 and Day 7.

Table 1 illustrates the ρ coefficient of Spearman correlation and R^2^ value of the linear regression between the ages of 144 donors and cytokine concentration calculated separately for Day 0 and Day 7. This analysis revealed that two cytokines were significant correlated with age but in reversed directions (Figure 4). MCP-1 levels were negatively correlated with age (Figure 4A), while eotaxin concentration was positively correlated with age (Figure 4B). The Spearman correlation coefficients ρ were -0.17 and -0.25 for MCP-1 and 0.2 and 0.17 for eotaxin (for Day 0 and Day 7 respectively). Even though the correlations were statistically significant, the R^2^ values calculated for linear regression were low, accounting for less than 4% of the variation, suggesting that the correlation between cytokine concentration and age does not necessarily fit a linear curve. PDGF-BB, IL-17A, G-CSF, IP-10 and VEGF had different concentrations between Day 0 and Day 7, but none of these cytokines were significantly correlated with age. 

**Table 1:** Correlation between age and serum cytokine concentration.

	**Day 0**				**Day 7**		
	**ρ**	**R^2^**	**p value**		**ρ**	**R^2^**	**p value**
**PDGF-BB**	**0.05**	4.84E-05	0.934		**-0.08**	5.33E-03	0.385
**IL-1b**	-0.16	2.10E-02	0.083		-0.21	2.64E-02	0.052
**IL-1Rα**	0.03	3.81E-04	0.816		0.007	1.03E-04	0.904
**IL-2**	not applicable				not applicable		
**IL-4**	0.12	7.92E-03	0.289		0.07	1.64E-03	0.630
**IL-5**	-0.01	2.33E-03	0.565		-0.008	2.96E-03	0.517
**IL-6**	-0.07	4.56E-03	0.421		-0.18	1.72E-02	0.117
**IL-7**	0.006	6.74E-05	0.922		0.01	1.46E-05	0.964
**IL-8**	0.05	6.78E-05	0.922		0.04	9.16E-03	0.254
**IL-9**	-0.01	5.04E-03	0.398		-0.13	3.04E-03	0.512
**IL-10**	-0.06	3.11E-02	0.334		-0.2	2.53E-04	0.850
**IL-12p70**	0.08	1.45E-05	0.964		0.007	2.58E-03	0.546
**IL-13**	0.07	2.28E-02	0.071		0.04	2.44E-03	0.557
**IL-15**	not applicable				not applicable		
**IL-17**	**0.08**	2.11E-03	0.585		**-0.02**	5.45E-04	0.781
**Eotaxin**	0.2	4.05E-02	0.0156*		0.17	3.62E-02	0.0224*
**FGF-b**	-0.23	2.61E-02	0.053		-0.27	3.73E-02	0.070
**G-CSF**	**0.08**	1.19E-03	0.681		**-0.03**	1.26E-03	0.673
**GM-CSF**	not applicable				not applicable		
**IFN-γ**	0.01	7.31E-03	0.308		0.01	9.25E-03	0.252
**IP-10**	**0.05**	4.96E-04	0.791		**-0.12**	5.02E-03	0.399
**MCP-1**	-0.17	2.94E-02	0.0399*		-0.25	3.63E-02	0.0223*
**MIP-1α**	-0.16	2.40E-02	0.064		-0.23	3.63E-02	0.062
**MIP-1β**	-0.06	1.42E-02	0.155		-0.21	3.51E-02	0.125
**RANTES**	0.1	2.37E-03	0.562		0.08	7.63E-04	0.742
**TNF-α**	-0.18	1.92E-02	0.098		-0.3	3.99E-02	0.106
**VEGF**	**0.05**	1.50E-03	0.645		**-0.03**	9.02E-04	0.721

Pvalue with * are significant (P<0.05)

In bold are highlighted spearman correlation that are different between Day 0 and Day 7

Results shown are ρ, R^2^ values and p values calculated from Spearman correlation and linear regression for both Day 0 and Day 7.

### 3.5 Serum cytokine concentrations and gender

We examined the correlation between gender and cytokine concentrations, and performed a Mann-Whitney test to evaluate differences in concentration between male and female. Cytokine concentrations for both Day 0 and Day 7 were averaged, and each analyte was reported for men and women (Table 2).

**Table 2:** Mean serum cytokine concentrations for Men and Women, and Caucasian vs Non Caucasian.

	**Gender analysis**			**Ethnicity analysis**		
	**Men Mean**	**Women Mean**	**p value**	**Caucasian Mean**	**Non Caucasian Mean**	**p value**
**PDGF-BB**	31617.7	27537.4	0.30	29111.8	33755.2	0.28
**IL-1b**	4.6	4.3	0.64	6.9	5.9	0.23
**IL-1Rα**	279.5	304.5	0.54	258.6	383.2	0.12
**IL-2**	nd	nd	na	nd	nd	na
**IL-4**	33.4	36.0	0.34	34.0	37.7	0.16
**IL-5**	5.8	8.0	0.18	6.2	7.1	0.39
**IL-6**	18.3	24.9	0.64	26.3	21.4	0.19
**IL-7**	52.2	59.5	0.60	50.8	81.0	0.17
**IL-8**	38.3	44.2	0.24	43.7	39.2	0.37
**IL-9**	113.6	573.3	0.13	168.4	1011.3	0.18
**IL-10**	2.7	2.7	0.94	5.6	5.5	0.95
**IL-12p70**	42.3	53.4	0.13	67.9	79.0	0.67
**IL-13**	11.2	14.1	0.10	17.3	17.0	0.90
**IL-15**	nd	nd	na	nd	nd	na
**IL-17**	208.2	201.5	0.79	180.7	260.4	0.05
**Eotaxin**	290.4	257.4	0.23	280.78	263.45	0.48
**FGF-b**	53.0	54.3	0.86	64.41	73.85	0.33
**G-CSF**	70.3	93.7	0.32	95.19	104.14	0.40
**GM-CSF**	nd	nd	na	nd	nd	na
**IFN-γ**	542.8	822.7	0.38	793.2	725.0	0.61
**IP-10**	2137.4	2238.1	0.58	2040.2	2573.3	**0.02**
**MCP-1**	62.8	55.4	0.23	68.0	42.2	**<0.001**
**MIP-1α**	13.2	15.1	0.36	20.1	18.5	0.61
**MIP-1β**	206.8	173.5	**<0.001**	191.7	174.4	0.18
**RANTES**	9534.5	11848.9	**<0.001**	12193.0	12171.0	0.98
**TNF-α**	37.0	68.7	0.52	138.4	50.5	**<0.001**
**VEGF**	246.0	263.3	0.50	247.1	297.3	0.10

p values in bold are significant (P<0.05)

nd= not detectable

na=not applicable

Results are shown as mean concentrations. P values shown in the table are calculated with non-parametric paired Mann-Whitney test using female serum specimens as controls, and Caucasian as control for ethnicity analysis.

Men and women differed significantly in the serum concentrations of two cytokines (*p<0.05*): the mean concentration of RANTES was higher in women than men (11848.9 pg/mL vs 9534.5 pg/mL, respectively [*p=0.0002*]) and MIP-1β, showed a mean concentration higher for men than for women (206.8 pg/mL vs 173.5 pg/mL, respectively [p=0.0012]). There were no significant differences in the remaining 25 cytokines, including the non-detectable ones.

### 3.6 Serum cytokine concentrations and ethnicity

 We examined the correlation between ethnicity and cytokine concentrations in Caucasian versus non-Caucasian, with the latter composed of Asian, African-American, Hispanic, and individuals of Middle Eastern descent. Cytokine concentrations for both Day 0 and Day 7 were average and each analyte was reported for the 2 ethnic groups (Table 2). Three cytokines showed significantly different serum concentrations (*p<0.05*): the mean concentration of MCP-1 and TNF-α were higher in Caucasian than non- Caucasian (68 pg/mL vs 42.2 pg/mL, (*p<0.001*) for MCP-1 and 138.4 pg/mL vs 50.5 pg/mL, (*p<0.001*) for TNF-α). IP-10, showed a mean concentration higher in non- Caucasian than Caucasian (2573.3 pg/mL vs 2040.2 pg/mL, respectively (p=0.02). The remaining 24 cytokines, including those undetectable, showed no significant differences.

## Discussion

In this study we measured the serum cytokine concentrations of 27 analytes using a Bio-Rad 27-plex Luminex kit at two time points in order to assess physiological cytokine variations in healthy volunteers. Since our cohort of volunteer donors had been pre-screened to eliminate those individuals with any acute or chronic illness, we did not expect to observe high levels of inflammatory or anti-inflammatory cytokines in these sera. The test results confirmed that in addition to low levels of the inflammatory and anti-inflammatory cytokines, the levels of IL-2, IL-15, and GM-CSF were undetectable. Samples in this study were stored in liquid nitrogen, in contrast to many clinical trials where such samples are stored in -80°C freezers. This was done for stability during long-term storage and to permit a wide assortment of assays to be performed on these samples in the future. We know of no deleterious effect of storing samples in this manner, and since all samples were stored in the same way, it is unlikely that storage contributed to the effects observed.

 We were interested in determining the temporal changes of cytokines and chemokines in the serum of healthy individuals. The results showed significant variations in the concentrations of only a few analytes in individual donors over time. The analysis of the standards showed technical stability in the detection of the cytokines, with all but two of the %CVs falling below 20% as seen in Table S1. The higher and lower limits of detection were consistent. Almost a third of the internal control bridge samples had %CVs above 20%, but all of these were much lower than the %CVs of the samples. 

We found that for 22 out of the 27 cytokines studies that the temporal effect of the intra-subject samples was not significant, although the variation among donors was still much larger and more significant than the intra-subject variability. We have no specific explanation of why the five cytokines, and only these five, showed such a high amount of variation over a 7-day period. RANTES, MCP-1, and MIP-1 have been reported to be type I cytokines, to act as coactivators of macrophages and together act functionally on NK cells [12]. These have also been reported to be elevated in lavages of allergic asthma patients [13]. Both PDGF-bb and VEGF are pro-angiogenic factors [14]. We speculate that the high fluctuations of these analytes likely represent sub-clinical, innate responses to minor immunologic or physiologic insults that are necessary to maintain homeostasis.

Only two cytokines (eotaxin and MCP-1) showed a relationship with increasing age, and increasing concentrations for eotaxin, and decreasing concentrations for MCP-1. Our finding for eotaxin is in agreement with earlier reports [15,16], but not for MCP-1. We also noted a difference in the levels of three cytokines between Caucasian and non-Caucasian subjects. Our finding for TNF-α and MCP-1 confirm previous findings associating Caucasians with higher levels of these cytokines [17,18]. 

Examining gender-related differences, our data also showed a significant difference in the cytokine concentrations between men and women for two cytokines, MIP-1β and RANTES, both of which are β-chemokines. RANTES was previously described as different between men and women in a study where PDGF-BB and RANTES were measured to describe gender differences in chemokine as marker of atherosclerosis [19]. Another study showed different fluctuations of MIP-1βconcentration in women, but not men affected with multiple sclerosis, [20], suggesting a relationship between gender and plasma levels of MIP-1β. This could also be due to the predominance of women in our cohort (65%). Hormonal menstrual cycles in the women volunteers were not evaluated, although with a median age of 27, most women were likely to be cycling or taking oral contraceptive. There has been a longstanding awareness of linkage between the endocrine and immune systems, as reviewed by Kennedy [21]. Previous studies [22] have documented increases in plasma cytokines related to phases of the menstrual cycle and the presence of estrogen receptors has been documented on a number of immune cells [23]. These estrogen receptors on immune cells have been implicated in up-regulation of cytokine production, which may help to explain the current findings.

All of these results taken together indicate that when using the measurement of cytokines as potential biomarkers it is important to be aware of the wide variation in cytokine levels not only between individuals but also within each individual over time. It is recommended that a sample from a subject prior to any treatment be used as a baseline control for that particular subject, rather than relying upon a matched control from a different individual. Furthermore, having two or more pre-intervention samples for each subject would permit understanding the intrinsic temporal variations that can occur without planned interventions.

## Supporting Information

Table S1 **Standard concentrations.**
(XLSX)

Table S2 **Detection limit for standard.**
(XLSX)

Table S3 **Internal control bridge sample.**
(XLSX)

Table S4 (XLSX)
