# Correction: Baseline Levels and Temporal Stability of 27 Multiplexed Serum Cytokine Concentrations in Healthy Subjects

**DOI:** 10.1371/journal.pone.0132870

**Published:** 2015-07-06

**Authors:** Angelique Biancotto, Abigail Wank, Shira Perl, Wendell Cook, Matthew J. Olnes, Pradeep K. Dagur, J. Christopher Fuchs, Marc Langweiler, Ena Wang, J. Philip McCoy

The authors wish to correct some errors with the data that came to light after the publication of the article:

Due to an error, the original Fig 2 shows the median values, rather than the mean values as is stated. A revised Fig 2 plotting the means is provided here.

**Fig 2:**
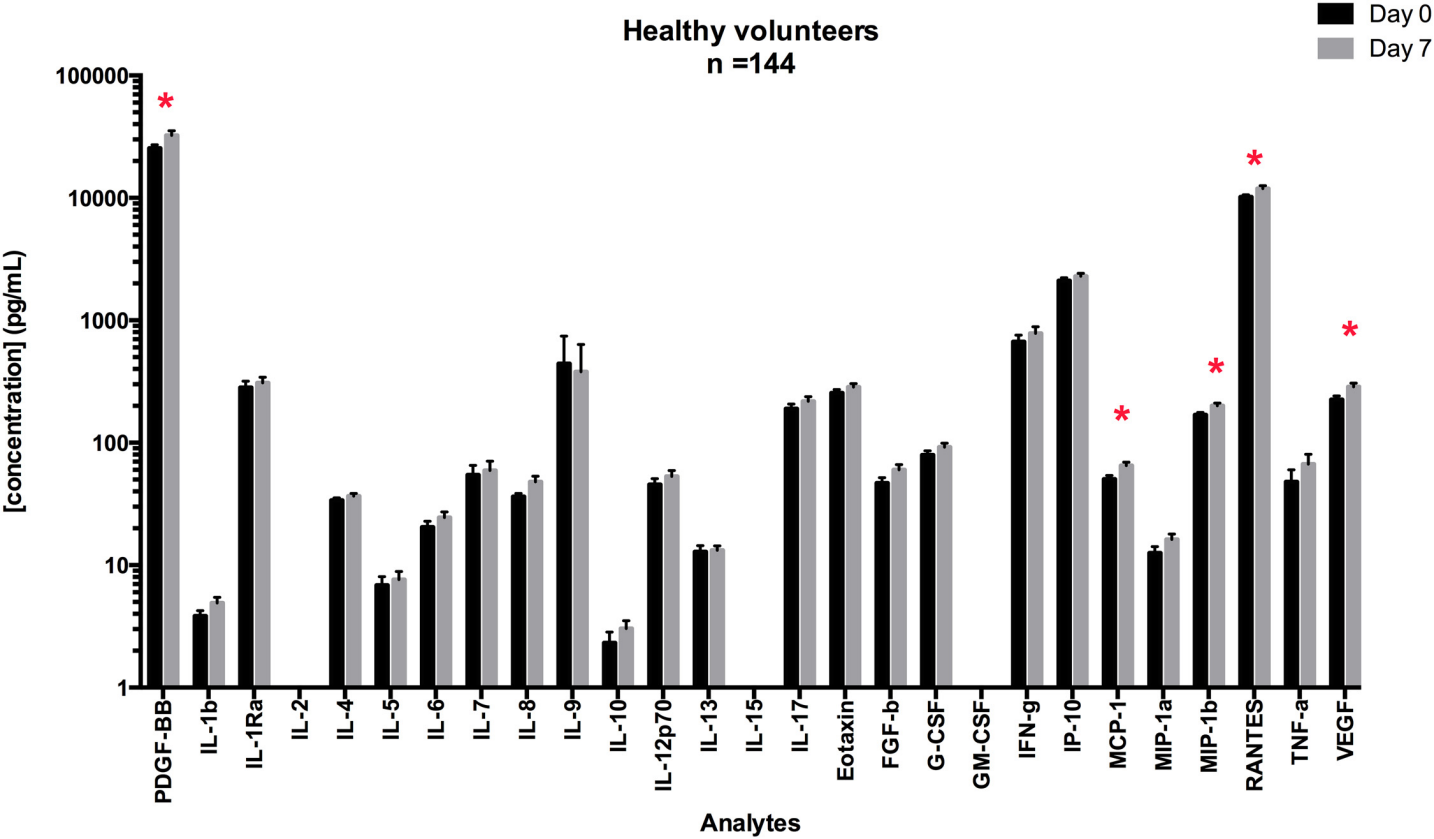
Mean concentrations for cytokines in serum samples at Day 0 and Day 7. Serum cytokine concentrations were measured on matched samples at Day 0 and Day 7. Paired T-tests were performed to measure the significance of the difference of the mean between Day 0 and Day 7. Cytokines for which %CV is higher than 20% are shown with “*”.

There was a discrepancy in the handling of data for Table 2. In the construction of the gender side of this table, we assigned values of "0" to any value that was out of range (OOR) low in order to allow statistical analysis. In the ethnicity side of the table, OOR readings were ignored in calculations rather than being assigned a value of "0". This led to inconsistencies in whole-population means between the ethnicity and gender analyses for cytokines where some values were OOR, but not for other cytokines where all values were in range. A revised Table 2 is provided in which all means have been calculated using a value of "0" where the reading was OOR low.

**Table 2:** Mean serum cytokine concentrations for Men and Women, and Caucasian vs Non Caucasian.

	Gender analysis			Ethnicity analysis		
	Men Mean	Women Mean	p value	CaucasianMean	Non caucasian Mean	p value
**PDGF-BB**	31617.7	27537.4	0.30	28529.5	29919.3	0.87
**IL-1b**	4.6	4.3	0.64	4.5	4.2	0.84
**IL-1Rα**	279.5	304.5	0.54	257.3	383.2	0.16
**IL-2**	nd	nd	na	nd	nd	na
**IL-4**	33.4	36.0	0.34	34.0	37.7	0.57
**IL-5**	5.8	8.0	0.18	6.5	8.9	0.58
**IL-6**	18.3	24.9	0.64	23.4	20.7	0.78
**IL-7**	52.2	59.5	0.60	48.0	77.3	0.42
**IL-8**	38.3	44.2	0.24	43.4	39.2	0.79
**IL-9**	113.6	573.3	0.13	150.7	1011.3	0.19
**IL-10**	2.7	2.7	0.94	2.8	2.5	0.87
**IL-12p70**	42.3	53.4	0.13	47.5	54.3	0.74
**IL-13**	11.2	14.1	0.10	13.0	13.3	0.94
**IL-15**	nd	nd	na	nd	nd	na
**IL-17**	208.2	201.5	0.79	178.9	260.4	0.13
**Eotaxin**	290.4	257.4	0.23	275.2	254.5	0.77
**FGF-b**	53.0	54.3	0.86	49.9	62.9	0.51
**G-CSF**	70.3	93.7	0.32	83.6	89.9	0.80
**GM-CSF**	nd	nd	na	nd	nd	na
**IFN-γ**	542.8	822.7	0.38	725.8	725.0	1.00
**IP-10**	2137.4	2238.1	0.58	2040.2	2573.3	0.20
**MCP-1**	62.8	55.4	0.23	66.0	39.9	0.09
**MIP-1α**	13.2	15.1	0.36	14.3	14.9	0.92
**MIP-1β**	206.8	173.5	**<0.001**	189.8	174.4	0.67
**RANTES**	9534.5	11848.9	**<0.001**	11095.7	10930.6	0.94
**TNF-α**	37.0	68.7	0.52	72.0	25.3	0.36
**VEGF**	246.0	263.3	0.50	239.7	297.3	0.37

p values in bold are significant (P<0.05)

nd = not detectable for more than 2/3 of samples measured

na = not applicable

Supplemental S4 Table also includes errors where some OOR values had not been replaced with "0" values. A revised version is provided here, as S4 Table.

The authors would like to apologize for these errors and confirm that these corrections do not affect the conclusions presented in the article.

## Supporting Information

S4 Table (XLSX)
